# Combined Left Central Retinal Artery Occlusion and Bilateral Anterior Ischemic Optic Neuritis: A Rare Presentation of Giant Cell Arteritis

**DOI:** 10.1155/2019/3236821

**Published:** 2019-09-22

**Authors:** Anne D. D. Joseph, Jebananthy Anandaselvam Pradeepan, Thirunavukarasu Kumanan, Muthusamy Malaravan

**Affiliations:** ^1^University Medical Unit, Teaching Hospital Jaffna, Jaffna, Sri Lanka; ^2^Department of Ophthalmology, Teaching Hospital Jaffna, Jaffna, Sri Lanka

## Abstract

Giant cell arteritis, a large vessel vasculitis is characterized by headache, visual impairment, constitutional symptoms, and increased inflammatory markers. Visual involvement in giant cell arteritis ranges from amaurosis fugax to permanent visual loss, and extensive bilateral visual impairment is a rare presentation. We hereby report a case of combined left central retinal artery occlusion and bilateral anterior ischemic optic neuritis in a patient who poorly responded to standard corticosteroid therapy.

## 1. Introduction

Giant cell arteritis (GCA), also known as temporal arteritis, is the most common systemic vasculitides in older adult populations [1, 2]. It is a disease often observed in the age group of more than 50 years with the peak incidence in the seventh decade. Many of the symptoms and signs of the GCA are due to the involvement of the cranial branches of arteries that originate from the arch of the aorta, but the vascular involvement can be widespread.

The most common symptom of GCA is a new onset headache, usually around the temporal region. Abrupt onset of visual disturbances, in particular transient monocular visual loss, jaw claudication, unexplained fever, fatigue, anemia, or other constitutional symptoms and signs with high erythrocyte sedimentation rate (ESR) and/or high serum C-reactive protein (CRP) are the common observations at presentation. Simultaneous onset of bilateral visual loss is an unusual clinical entity.

## 2. Case Presentation

A 75-year-old lady, known patient with optimally controlled hypertension with amlodipine monotherapy for 5 years duration, presented with sudden painless onset of visual blurring involving the left eye for 3 days duration which was progressed to the level of perception of light (Pl) on the same side. Approximately 24 hours later, she developed gradual blurring of vision in the right eye too. She had left-sided intermittent jaw pain for a week prior to the onset of visual symptoms; however, she did not have a history of fever, frontotemporal headache, malaise, or constitutional symptoms. Past ophthalmological history included bilateral uncomplicated cataract extraction two years prior to the current presentation, and her visual acuity was 6/12 in the left eye and 6/12 in the right eye prior to the admission.

On examination, she was oriented and alert with the Glasgow Coma Scale (GCS) of 15 out of 15, was afebrile, and had tenderness over the left temporal region, but no thickening of the temporal artery was noted. Ophthalmic examination showed her visual acuity in the left eye Pl; in right eye, it was 1/10 with complete ocular motility. Other system examinations including the neurological examination were unremarkable.

**Figure 1:**
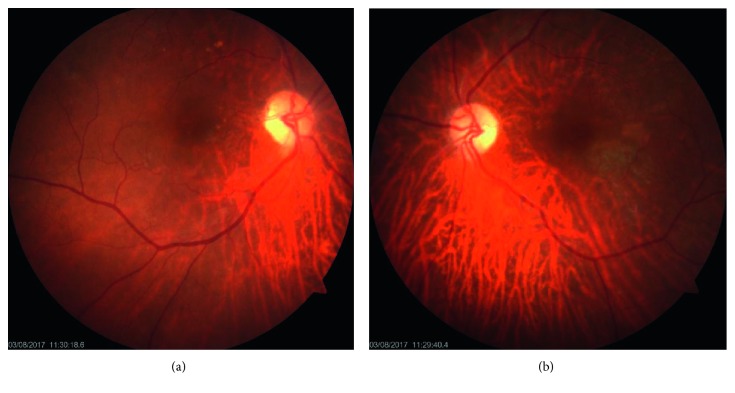
Fundus photograph of the right (a) and left (b) eyes. The right eye shows a pale disc, and the left eye shows a pale disc and cherry red spot.

Detailed examination of the eyes by the ophthalmology team revealed both pseudophakic eyes with normal intraocular pressures in each eye (right eye 12 mm·Hg and left eye 14 mm·Hg). She had a bilateral pale disc and evidence of central retinal artery occlusion in the left eye (Figure 1).

Investigations revealed ESR 130 mm/1^st^ hour, CRP 101 mg/L, white blood cell count 9560/mm^3^ with neutrophil predominance, haemoglobin 9.7 g/dL with normochromic normocytic cells, and platelets 350000/mm^3^ Her urine analysis, renal, and liver profile were normal. Chest radiograph, electrocardiogram, and 2D echocardiogram were unremarkable. As she had visual impairment, a magnetic resonance imaging (MRI) of the brain was performed, which also was unremarkable.

A clinical diagnosis of giant cell arteritis was made and was proceeded with a left side temporal artery biopsy which confirmed the diagnosis of giant cell arteritis. She was initiated on intravenous methylprednisolone 1 g daily for 3 days on presumptive diagnosis, while the histological confirmation was awaited and followed by 40 mg prednisolone (1 mg/kg) oral daily for four weeks.

A negative result obtained in aetiology directed investigations including anti-nuclear antibodies (ANA), venereal disease research laboratory (VDRL), retroviral screening, and tuberculosis quantiferon gold test.

After continuation of tapering dose of glucocorticoids for a period of 6 months, she noted to have no significant improvement of vision in both eyes. But her vision was not deteriorated after the steroid therapy.

## 3. Discussion

Giant cell arteritis is a large vessel vasculitis. It mainly involves the aorta and its branches. Visual impairment in GCA can be transient described as amaurosis fugax or permanent. Bilateral visual impairment is a rare presentation in this spectrum [2–4].

Even with the prompt corticosteroid therapy, permanent, complete, or partial visual loss in one or both eyes is reported to be 15 to 20%, and involvement of both eyes is rare and the incidence is less than 1% [3, 5]. Common fundoscopic finding in GCA is anterior ischemic optic neuropathy (AION), and also they can have central retinal artery occlusion, central retinal vein occlusion, or branch artery occlusion [3, 5]. This is the first case reported from Sri Lanka that describes a bilateral AION and central retinal artery occlusion which is an unusual extensive ophthalmic involvement of GCA [3, 5].

Even though the onset of headache is a striking feature, this particular patient had neither headache nor significant constitutional symptoms at presentation. She had biopsy-proven GCA, supported with clinical features such as age more than 50 years, elevated ESR (>50), and tenderness over the temporal artery region. The presentation itself is 93.5% sensitive and 91.2% specific to diagnose GCA according to the American College of Rheumatology (ACR) criteria. She had jaw claudication which is considered as an additional adverse prognostic marker that predicts visual impairment [5]. Additional aetiology-directed investigations were done to exclude the other possible causes of central retinal artery occlusion.

With the initial presentation of visual loss, the standard therapy recommended is high-dose steroids of intravenous methylprednisolone 500 mg–1 g daily for three days followed by oral prednisolone in a dose of 1 mg/kg/day (maximum 60 mg/day) [6, 7]. The blindness caused by GCA is preventable in the majority of cases when diagnosis is made early in the course of the disease [8] as the delay in diagnosis or treatment of GCA can cause several serious consequences, including irreversible visual loss.

However, in this case immediate high-dose steroid therapy was initiated ahead of temporal artery biopsy, but did not result in a favorable outcome in terms of vision though no progressive visual deterioration was noted after initiation of treatment as described in some studies [5, 8, 9].

In conclusion, this case demonstrates an extremely rare spectrum of clinical presentation with an ophthalmic involvement of giant cell arteritis. Even with standard glucocorticoid therapy, the outcome in terms of vision was minimal in this particular patient. When an elderly individual presents with striking symptoms of transient visual loss, headache, and jaw claudication, GCA should be considered as an important differential diagnosis to prevent undue delay in initiating steroid therapy. Jaw claudication was the only initial symptom of this patient which was overlooked by the patient and resulted in a preventable serious lifelong disability.
